# The Relationship Benefits of Auto Maintenance and Repair Service: A Case Study of Korea

**DOI:** 10.3390/bs10070115

**Published:** 2020-07-12

**Authors:** Jinpyo Hong, Boyoung Kim, Sungho Oh

**Affiliations:** Seoul Business School, Seoul School of Integrated Sciences and Technologies (aSSIST), Seoul 03767, Korea; kia1@stud.assist.ac.kr (J.H.); multiosh@gmail.com (S.O.)

**Keywords:** auto maintenance and repair service, relationship benefits, customer engagement, relationship orientation, service trust, service satisfaction

## Abstract

This study aimed to empirically examine what effects confidence, social, and economic benefit factors have on continuous relationship orientation through the mediation of service trust, service satisfaction, and customer engagement factors in the auto maintenance and repair service sector. This study carried out a questionnaire survey with 319 customers using auto maintenance and repair service and verified hypotheses. As a result of the analysis, the confidence and social benefits of auto maintenance and repair service affected service trust, while the confidence and economic benefits affected service satisfaction. Service trust did not affect customer engagement or long-term relationship continuity but affected them when it mediated service satisfaction. Consequently, it was revealed that confidence benefit should be consolidated and that professionalism or service quality excellence in maintenance or repair becomes the most important factors to produce customer engagement or long-term relationship continuity in the auto maintenance and repair service. Although it is vital to improve trust or service, it is confirmed that a relationship can be maintained only if the auto maintenance or repair service is satisfactory.

## 1. Introduction

Most service companies offer new services and benefits to continuously maintain relationships with existing customers or create higher customer satisfaction according to the individualization of customer needs and differentiated service demand increase. Since service has intangible characteristics, broader customer contacts are made in the process of meeting customer expectations, and interactions with customers produce a positive impression or trust, which works as a key factor for relationship continuity. In the end, relationship benefits revealed through interactions between companies and customers play a pivotal role in retaining customers. The after-sales service, one of the methods for achieving customer satisfaction in auto maintenance and repair service, is most valuable, and efficient after-sales service becomes the highest priority of auto maintenance and repair service companies [1]. However, customers can personally manage auto maintenance and repair nowadays, including accident history and consumable parts replacement cycle based on smartphones, through new systems improving auto maintenance and repair management as advanced technologies are applied. Furthermore, general visit-management services, such as car wash, light maintenance, and repair and consumable parts replacement by visiting, due to platform invigoration, have continuously developed. 

In the changed maintenance and repair service market environment, the need for after-sales service satisfaction enhancement, differentiated service offering for customer retainment, and customer relationship maintenance strategy increases. For example, BMW additionally offers the BMW Service Inclusive (BSI), warranting consumable parts replacement and regular inspection within 100,000 km for five years, in addition to basic warranty service. Toyota renders efforts to diversify customer service through such assistances as the “Express Service” by which inspection can be finished within an hour. Meanwhile, Lexus offers a 24-h emergency mobilization service and provides a female lounge strictly for female customers. Hyundai Motors and Kia Motors push ahead with an excessive maintenance and repair prevention program to compensate up to 300% of excessive charge if an excessive maintenance and repair service is presented to the customer service center after the excessive service, and when it is judged so after an expert’s investigation. 

Such efforts of the companies in the auto maintenance and repair service show that existing customer retention and relationship consolidation is a more important task, rather than securing new customers, due to a rapid change of technology and market environment and an increase in customer needs and service expectation level. As previous studies [2,3] assert that income can increase from 25% to 85% if customer churn rate is reduced by 5%, the retention of existing customers and relationship consolidation through the establishment of customer relationships become a key marketing strategy direction to auto maintenance and repair service companies. For their steady earnings creation and efficient management, it is important to strengthen long-term relationships with customers, and two-way communication and close relationship establishment are important because companies and customers have a win-win relationship for mutual value, and not a mutually competing relationship [4]. One of the long-term strategies forming, maintaining, and consolidating relationships between companies and customers is relationship marketing. If a company has customers for the long-term, the company’s earnings can be assured to some degree. Relationship marketing brings about positive results, including customer participation and efficient customer responses [5].

Even though various studies on relationship benefits, relationship quality, and customer retention between companies and customers have been actively performed [6], few studies on relationship benefits or relationship quality between auto maintenance and repair service companies and customers in which relationship continuity with customers has important industrial characteristics have been carried out. In this context, this study purposed an empirical analysis of the effects of the relationship benefits on long-term relationship establishment in customers, using perception to auto maintenance and repair service with meditated effect of service trust, repair service, and customer engagement attributes. In the auto maintenance and repair service process, a service provider’s role is important, in addition to service quality, and there is a need to induce customers’ active re-purchase and intention to orally pass down the service provider through customers’ positive feelings and experiences at the service contact point. From this aspect, this study presented specific implications for service process improvement and marketing strategy within the auto maintenance and repair service industry. 

## 2. Theoretical Background and Hypothesis Development

### 2.1. Relationship Benefits, Service Trust, and Service Satisfaction

In the service process, a service provider’s role is essential, in addition to service quality. At the service contact point, customers’ positive feelings on and experiences with service providers can be connected to their re-purchase and positive intention to pass down feedback on the service providers [7]. Reichheld et al. [8] asserted that companies can increase 100% of their profits by maintaining more than 5% of their customers. Customer churn can be connected to corporate profit increase through long-term relationship retention with customers. From this perspective, relationship benefits mean the benefits that a company can offer to customers if the company’s understanding of the customers is enhanced once a firm’s relationship with customers is maintained for a certain period of time through relationship development. Gwinner et al. [9] defined all types of benefits offered to customers as the concept of relationship benefits. Palmatier et al. [10] insisted that relationship benefits are one of the key strategies to ensure the service company’s profitability and competitive advantage. Companies need to maintain a relationship with customers for a certain period of time, and their understanding of customers is enhanced through the process, where the company can finally offer the benefits that customers want [11]. 

In many studies, the social, psychological, and individualized relationship benefits of Quach et al. [12] are slightly differently categorized by researchers. Reynolds and Beatty [13] categorize relationship benefits into confidence, social, and special treatment benefits, while Ulaga [14] classifies relationship benefits into benefits, procedures, and operational benefits. Conze et al. [15] define relationship benefits as psychological, social, special treatment, and diversity-pursuing benefits. Reimer and Kuehn [16] performed a study with economic, social, psychological, special treatment, and information benefits. This study was performed with confidence and social benefits existing between the service providers and customers and with economic benefits that become the most basic in relationship benefits in consideration of auto maintenance and repair service characteristics with low customer contribution to the limitation and function of the special treatment category. Confidence benefit reduces worries and makes customers feel comfortable, as it can predict achievement with the feeling of belief in service providers [17]. Social benefit makes customers feel an affinity towards the service providers, enabling social relationship [18]. Lastly, economic benefit is what customers receive based on time and cost savings or functional convenience [19]. 

By continuously providing relationship benefits based on customer preference, relationship benefits form a significant relationship quality with customers [20]. Darkhantuya [21] said that relationship benefits have a partially significant effect on customer satisfaction, and Kang and Kim [22] also reported that relationship benefits have a partially significant effect on customer satisfaction. The formation and retention of long-term relationships with customers create a significant effect on relationship quality, customer satisfaction, and customer loyalty through strong service [23]. Consequently, relationship benefits are revealed among trust, commitment, and satisfaction [24]. This study tried to grasp the association between service relationship benefits and relationship quality with two factors, that is, service satisfaction and service trust formed in customer relationship among relationship quality factors. This study set the following hypotheses on customer trust on and satisfaction with auto maintenance and repair service and with regard to confidence, social, and economic benefit factors:

**Hypothesis 1** **(H1).**  *Confidence benefit of the relationship benefits on auto maintenance and repair service will have a positive effect on service trust.*

**Hypothesis 2** **(H2).**  *Social benefit of the relationship benefits on auto maintenance and repair service will have a positive effect on service trust.*

**Hypothesis 3** **(H3).**  *Economic benefit of the relationship benefits on auto maintenance and repair service will have a positive effect on service trust.*

**Hypothesis 4** **(H4).**  *Confidence benefit of the relationship benefits on auto maintenance and repair service will have a positive effect on service satisfaction.*

**Hypothesis 5** **(H5).**  *Social benefit of the relationship benefits on auto maintenance and repair service will have a positive effect on service satisfaction.*

**Hypothesis 6** **(H6).**  *Economic benefit of the relationship benefits on auto maintenance and repair service will have a positive effect on service satisfaction.*

### 2.2. Service Trust, Service Satisfaction, and Customer Engagement

As for studies related with relationship benefits in a variety of fields that targeted service companies, papers researching the effects on relationship achievements such as loyalty and passing down feedback through relationship quality, including customer satisfaction, trust, and commitment are the mainstream research trend [25]. Crosby et al. [26] presented relationship quality as the degree of interactions between sellers and purchasers and reported that relationship quality consists of trust and satisfaction. Mohr and Spekman [27] reported that a successful partnership is composed of commitment, trust, coordination, participation, communication quality, and common problem resolution. Storbacka et al. [28] presented a dynamic model of relationship quality and asserted relationship quality as satisfaction, communication, commitment, and solidarity. Harris and Ezeh [29] conceptualized relationship quality into three structures composed of customer trust, achievement of work required in relation to work performance, and customer commitment to a corporate relationship. Van Doorn et al. [30] defined relationship quality as the suitability level of relationship in meeting customer needs, which is similar to the concept of product quality. 

As customer service experience or participation changes vigorously and actively, the concept of customer engagement emerges beyond commitment. Customer engagement is a customer’s active participation toward companies as derived from motive stimulation beyond purchase [31]. Brodie et al. [32] defined customer engagement as a psychological state generated from mutually and jointly creative customer experience with companies in a relationship between companies and customers. Customer engagement consists of interest, interaction, and commitment as it is revealed as an interaction between customers. And it reveals oral passing down activity, posting on blogs, co-participation, and customer evaluation on experienced goods or services [33]. 

Based on previous studies, customers satisfied with the current auto maintenance and repair service can change their selection if there are products or services that offer higher satisfaction [34]. This study tried to examine service trust, which is a psychological belief state indicated in the exchange relationship between customers and car maintenance and repair centers. Moreover, this study designed a hypothesis that customer’s trust in auto maintenance and repair service will have a positive effect on satisfaction. In addition, this study designed and proved the following hypotheses from the following aspects: given that customer interest in auto maintenance and repair service increases and market environment changes with higher active participation, customers will show their emotional and voluntary behaviors toward companies based on a fair relationship with companies in the auto maintenance and repair service industry [35,36].

**Hypothesis 7** **(H7).**  *Trust in auto maintenance and repair service will have a positive effect on customer satisfaction with the service.*

**Hypothesis 8** **(H8).**  *Trust in auto maintenance and repair service will have a positive effect on customer engagement.*

**Hypothesis 9** **(H9).**  *Satisfaction with auto maintenance and repair service will have a positive effect on customer engagement.*

### 2.3. Service Trust, Service Satisfaction, and Long-Term Relationship Orientation

Long-term relationship orientation can be a firm’s prime goal to maintain relationships with existing customers for a long time and prevent churn to other companies [37]. Previous studies on long-term relationship orientation are discussed in various approaches. From mutual benefit between transaction parties, Kelley and Thibaut [38] said that common achievements of transaction parties including suppliers are the mutually dependent common activity results over a long period of time. Gwinner et al. [9] reported that purchasers reduce transaction experiences or future benefit’s uncertainty by forming a long-term bond with suppliers to obtain specific benefits that cannot be obtained from a short-term transaction relationship; therefore, long-term relationship orientation between purchasers and suppliers is sought. 

Long-term relationship orientation is based on how relational the existing transactions have been, rather than on the period of forming relationships between customers and companies beyond a simple, repeated behavioral purchasing. Johnson et al. [39] explained that both parties forming a transaction relationship to meet end-user needs assert their own activities from a long-term perspective, while they explained a partnership-like, thinking-dominating transaction relationship by which one party’s success can be decided by the other party as long-term relationship orientation. Long-term relationship orientation is a concept encompassing relationship continuity and interdependence between companies and consumers, and it is the concept containing attitude and behavioral intention. Therefore, it can include repeated purchasing behaviors, an intention to pass down feedback, and an intention to continue the relationship [40]. 

The long-term relationship orientation is revealed as a consumer’s conscious judgment or evaluation result and is affected by perceived psychological factors. Consequently, it is linked with factors like customer engagement affecting customer satisfaction with service, trust in products, or services, and customer’s active relationship improvement according to experiences as mentioned in previous studies [41,42,43,44]. Flavián et al. [45] asserted each party’s activities from the long-term perspective between the parties in a transaction relationship to meet customer needs, stating that both parties should perceive the other party as in a partner relationship. It means that factors such as trust, dependence, environmental uncertainty, reputation, and satisfaction affect long-term relationship orientation. Lai [46] said that trust in and satisfaction with salespersons are major factors that consumers consider when forming a continuous relationship with sellers. 

And relationship quality improvement, including satisfaction and trust between companies and customers in service sales and experience activities between companies in various service industries and customers, has a significant effect on long-term relationship orientation [47,48,49]. Consequently, service trust and service satisfaction as relationship qualities regarding auto maintenance and repair service felt by customers will have a significant effect on long-term relationship orientation.

**Hypothesis 10** **(H10).**  *Trust in auto maintenance and repair service will have a positive effect on long-term relationship orientation.*

**Hypothesis 11** **(H11).**  *Satisfaction with auto maintenance and repair service will have a positive effect on long-term relationship orientation.*

Because customer engagement is a customer’s relationship-forming behavior to create and maintain a relationship between companies and customers through a customer’s voluntary and positive participation, such customer behavior can be revealed as long-term relationship orientation with companies [50,51]. This study designed a hypothesis that customer engagement in auto maintenance and repair service will have an effect on long-term relationship orientation with customers.

**Hypothesis 12** **(H12).**  *Customer engagement improvement on auto maintenance and repair service will have a positive effect on long-term relationship orientation.*

## 3. Research Methods

### 3.1. Research Model

**Figure 1:**
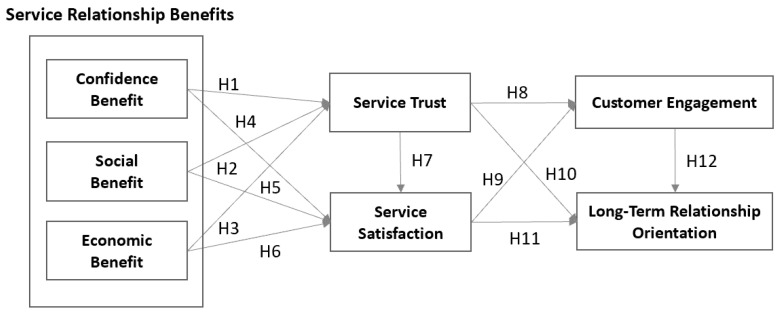
Research model.

Through the hypotheses drawn based on previous studies, a study model as shown in Figure 1 was designed. Confidence, social, and economic benefits, which can be defined as relationship benefits in auto maintenance and repair service, were set as independent variables. As parameters, service trust and service satisfaction were set; thus, whether the relationship benefits had effects on the dependent variables, customer engagement, or long-term relationship orientation through the mediation of two factors, which were service trust and service satisfaction, was set. This study set each path composition to check whether service trust has the effect on service satisfaction in the auto maintenance and repair service customer group and to check whether customer engagement has an effect on long-term relation orientation. 

### 3.2. Measurement Variables 

For data collection to analyze the model, a questionnaire survey was carried out; the questions were composed through previous studies, as shown in Table 1, and manipulative variables of the questionnaire components to be composed of questions were defined. The variables defined as above consisted of questions as shown in Table 1 and were investigated with a 5-point Likert scale (1 = strongly disagree, 2 = disagree, 3 = no opinion, 4 = disagree, 5 = strongly agree). Three questions were composed, each for confidence benefit and social benefit based on the studies of Gwinner et al. [9], Reynolds and Beatty [13], and Henning-Thurau et al. [25]. Likewise, there were three questions for the economic benefit based on the studies of Yen and Gwinner [17] and Koritos et al. [19]. Two questions were composed, each for service trust and service satisfaction based on a study of Sirdeshmukh et al. [52] and the studies of De Wulf et al. [53] and Eggert and Ulaga [41], respectively. For customer engagement, two questions were composed in order to be passed down orally and with an intention to participate for improvement based on the studies of Brodie et al. [32] and So et al. [54]. Lastly, three questions were composed for long-term relationship orientation based on the studies of Ganesan [55] and Jahanshahi et al. [56]. The item “Not anxious when using the service” in confidence benefit and the item “Receive much discount” in economic benefit were excluded in this study as they were not significant as a result of the analyses of measurement model trust and convergent validity. 

### 3.3. Survey and Analytic Methods

The questionnaire survey targeted customers having experiences of using auto repair centers. In consideration of car use and the number of maintenance and repair service, the customers using auto maintenance and repair service in Seoul and major cities in Gyeonggi Province in South Korea were selected. The copies of the questionnaire that were drawn up with Google Survey for 30 days from July 1 to July 30, 2019, were distributed and collected through email and SNS (social network sites). Finally, 464 questionnaire responses were collected, and a total of 319 were analyzed, except for 145 insincerely responded to questionnaire copies. For data analysis, SPSS 24.0 was used and basic data reliability and validity were examined through demographic characteristics, descriptive statistics, and exploratory factor analyses. The factor analysis and model verification, along with path analysis for structural equation model analysis, were analyzed using AMOS 25.0. For the mediation effect verification of service trust and satisfaction, a bootstrapping technique in line with the previous study guide of Gallagher et al. (2008) was used. 

## 4. Results

### 4.1. Demographic Information of the Data

As a result of carrying out a questionnaire survey targeting auto maintenance and repair service-experiencing customers, the demographic analysis result for 319 customers is shown in Table 2. There were over three times more males (79.3%) than females (20.7%). As for age, the customers in their 40s, 30s, and 50s were 33.2%, 29.2%, and 26%, respectively, and those in their 40s showed the highest experience ratio of car repair centers. Concerning customers’ occupation categorization, service industry showed 25.7% and manufacture/production showed 19.1%, while others took up a high ratio, which shows that the customers were distributed to various occupation groups. 93.4% of the customers visited car repair centers for the maintenance and repair of their cars, with the interest in car maintenance and repair being as follows: very high showing 52.4% and moderate being 40.4%, which implies chiefly high interest. 

### 4.2. Analysis Results of Reliability and Validity 

For the analysis of the reliability and validity of the structural equation measurement model, it can be said that internal consistency reliability was ensured if the composite reliability index was 0.7 or higher [57]. Convergent validity is evaluated with factor loading, Cronbach’s α, and composite reliability index, and it can be said that convergent validity is ensured if factor loading is 0.4 or more, Cronbach’s α is 0.6 or more, and statistical significance is shown [58]. In line with the above criteria, factor loading was 0.645–0.851, all were more than 0.6, and thus they were good, whereas internal consistency reliability ensured significance with 0.759–0.855 (composite reliability). Because the t value of all was 6.0 or more, statistical significance was confirmed. The AVE (average variance extracted) value was 0.616–0.720 and Cronbach’s α value was 0.713–0.789; therefore, convergent validity was ensured. As a result of analysis on the measurement model fit, χ^2^(p) was 76.066 and χ^2^/degree of freedom was 1.729. Goodness-of-Fit-Index (GFI) value was 0.968, Adjusted Goodness-of-Fit Index (AGFI) was 0.944, Normal Fit Index (NFI) was 0.961, and Root Mean Square Error of Approximation (RMSEA) was 0.043. The composition values of the measurement model fix were excellent (see Table 3). 

In the case of correlation analysis, as shown in Table 4, it can be said that discriminant validity is ensured among potential variables if the square root value of AVE calculated between potential variables in line with the criteria presented by Gallagher et al. [59] is larger than each potential variable’s correlation coefficient. As a result of the analysis on the AVE values and correlation coefficients among potential variables in Table 4, each potential variable’s AVE square root value was larger than the correlation coefficient among potential variables, whereas the correlation coefficient values were 0.7 or more and were significant; therefore, discriminant validity was ensured.

### 4.3. Analysis Results of Structural Model 

As shown in Table 5, prior to the path analysis, χ^2^(p) was 291.571, χ^2^/degree of freedom was 2.804, GFI value was 0.900, namely 0.9 or more, whereas AGFI was 0.853, NFI 0.954, and RMSEA 0.075, and goodness-of-fit component values were excellent, and therefore the goodness of fit was significant. CFI representing a model’s explanation power was 0.970, TLI judging the explanation power of the structural model was 0.960, and all were 0.9 or more, and thus the basic model was analyzed to be very fitting. As a result of the path analysis through the structural equation modeling analysis, three hypotheses out of 12 hypotheses were rejected. 

Table 6 shows the direct and indirect effects analysis result. The confidence benefit (10.952) and social benefit (3.331) of the service relationship benefit factors showed a positive effect on service trust, and thus the hypothesis was accepted. However, the economic benefit did not have an effect on service trust. The confidence benefit (5.104) and economic benefit (2.449) had a positive effect on service satisfaction, but the social benefit factor rejected the hypothesis. Meanwhile, service trust did not have an effect on customer engagement or long-term relationship orientation. In contrast with this, service satisfaction had a positive effect on customer engagement (4.041) and long-term relationship orientation (3.090); thus, the hypothesis was accepted. It was confirmed that service trust had a positive effect on service satisfaction (6.048) in auto maintenance and repair service as shown in previous studies [60,61] and that customer engagement had a positive effect on long-term relationship orientation (7.764), therefore rendering the hypothesis acceptable. 

As a result of mediation effect verification on service trust and service satisfaction factors using the bootstrapping technique, the service trust mediated confidence benefit (0.285**) and social benefit (0.114**) but did not mediate economic benefit. Specifically, there is a need to consolidate confidence and social benefits rather than economic benefits to enhance satisfaction through service trust. The service trust (0.233*), confidence benefit (0.536**), social benefit (0.156**), and economic benefit (0.139*) that used service satisfaction on customer engagement as mediation showed indirect effects, thus confirming to work as parameters. The indirect effects of all variables on long-term relationship benefit also showed significant difference and, therefore, all factors including service satisfaction and service trust were confirmed to show mediation effects. This reveals that service trust can show effects through the mediation of service satisfaction, although service trust cannot directly affect customer engagement and long-term relationship organization. 

## 5. Conclusions

This study aimed to verify the effect relationship between service relationship benefits and service trust, service satisfaction, customer engagement, and long-term relationship orientation by targeting auto maintenance and repair service customers in order to seek methods to retain customers and enhance relationship continuity through the customer relationship marketing invigoration of auto maintenance and repair companies. As a result of the analysis, three conclusions were drawn. First, the economic benefit out of the confidence benefit, social benefit, and economic benefit factors, which were the relationship benefit factors of auto maintenance and repair service, did not have an effect on customers’ service trust. The customers’ service satisfaction was affected through the economic benefit in auto maintenance and repair service as previous studies [57,62] presented economic benefit such as prices or discount rates as an important factor to service consumption behaviors of customers. Furthermore, the analysis result was confirmed that service trust was affected by social benefits, rather than by economic or confidence benefits. It means that the social benefit of service relationship in auto maintenance and repair service is significant to lead a strong relationship with customers. 

Second, the social benefit of auto maintenance and repair service had an effect on service trust but did not have an effect on service satisfaction. Through the result, it was confirmed that affinity or relationship with repair center workers affected trust formation, but it did not directly affect the service satisfaction with auto repair or maintenance result, and that such direct factors as service quality or cost-effectiveness, namely economic and confidence benefits, were more important. It opposed to the previous researches identified the relationship benefits have a positive effect on service satisfaction [60,61]. It means that the customer’s behavior toward the service center and the relationship with workers in the auto maintenance and repair service sector should be objective and reasonable rather than other service sectors. 

Third, as a result of a mediation effect analysis, service satisfaction showed significant mediation effects on customer engagement and long-term relationship orientation with regard to service relationship benefit factors. Meanwhile, service trust did not show any direct mediation effect but showed the mediation effect through double mediation on service satisfaction. This means that auto repair centers need to concentrate on service satisfaction to directly affect customers’ behavior interest or long-term relationship formation, although service trust is important to the customers using auto maintenance and repair service. Through the result, it was confirmed that a strategy to improve service satisfaction based on trust rather than concentration on service trust establishment for customer management is more important to the auto repair centers, unlike in the previous studies [63,64,65,66], asserting that establishing loyalty and transaction continuity by gaining service trust can be a positive customer management strategy to general service companies. 

Consequently, it was verified that the consolidation of confidence benefit, namely maintenance and repair professionalism and expertise and quality excellence on service result, was a highly important factor that induced customer trust and satisfaction, and thus long-term relationship continuity in auto maintenance and repair service. This was a characteristic that the auto maintenance and repair industry had, which showed the importance of maintenance and repair works for relationship continuity, although social benefits or trust formation was valuable according to the characteristics of service attributes associated with safety or management professionalism, unlike customer activity or experience-oriented services such as restaurant, banking, and tourism. 

Auto maintenance and repair service companies need to emphasize maintenance and repair service professionalism and excellence in building up interactions with customers or a communication strategy for long-term customer relationship marketing. They also need to seek service differentiation by establishing marketing contents linked with auto management and maintenance and repair technological capabilities and systems. From this aspect, this study has significance in that it specifically presented a relationship marketing strategy direction by examining various effect relationships such as service trust, satisfaction, and customer engagement, as well as relationship benefit factors on auto maintenance and repair service beyond service quality and satisfaction factors dealt with in the previous studies. 

Nonetheless, this study has the following research limitations. First, this study targeted auto maintenance and repair service-experiencing customers within South Korea and, therefore, there is a research limitation that the study result cannot be generalized as maintenance and repair service relationship benefit characteristics since the Korean auto maintenance and repair service market and customer characteristics have been reflected. A further study needs to research this by expanding the target market to the Asian, American, and European areas, thus drawing the relationship benefits and customer behavior of auto maintenance and repair service targeting the global market, and comparatively research according to each continent’s characteristics. 

Second, this study has a research limitation in that it did not apply the relationship benefit factors that the auto maintenance and repair service had by applying service companies’ relationship benefit factors to the auto maintenance and repair service companies. A further study needs to examine more specific relationships and customer behavior factors based on the research in order to draw and define unique relationship factors within the repair centers and customers in the auto maintenance and repair service industry. Furthermore, future research should investigate the direct effect between the relationship benefits and long-term relationship orientation even if this research did not suggest the hypothesis of it. 

Lastly, various services exist and have been segmented in the auto maintenance and repair service including test driving upon car purchase, maintenance/repair and warranty repair according to car use, used car disposition and inspection, scrap car handling, and parts purchase. Therefore, research needs to be developed by considering the diversity of the auto maintenance and repair service process and segmenting auto repair company size, facility conditions, and customer’s service-experience level. 

## Figures and Tables

**Table 1:** Measurement variables and items.

Variable	Survey Item	Item	References
Confidence benefit	- Have a firm belief in service quality - Have a conviction in service procedure professionalism - Not anxious when using the service	3	Gwinner et al. [9] Reynolds and Beatty [13] Henning-Thurau et al. [25]
Social benefit	- Maintain friend-like relationship - The service provider recognizes me - Feel joy when receiving the service	3	
Economic benefit	- Use the service at cheap price - Receive the service at reasonable price - Receive large price discount	3	Yen and Gwinner [17] Koritos et al. [19]
Service trust	- Always believe in and visit the car repair center - Have a belief that the center will do its best for customers	2	Sirdeshmukh et al. [52]
Service satisfaction	- Satisfied with the service overall - Satisfied with workers’ working mode and repair	2	De Wulf et al. [53] Eggert et al. [41]
Customer engagement	- Have an intention to publicize the repair center using SNS - Have an intention to suggest an improvement for the service	2	Brodie et al. [32] So et al. [54]
Long-Term relationship orientation	- Will continue to visit the repair center - Have no intention to change the repair center - Will maintain long-term relationship with the repair center	3	Ganesan [55] Jahanshahi et al. [56]

**Table 2:** Demographics of survey participants.

Classification		Frequency	Percentage (%)
Sex	Male	253	79.3
	Female	66	20.7
	Total	319	100
Age	Under 30 years old	37	11.6
	30s–40s	93	29.2
	40s–50s	106	33.2
	Over 50 years old	83	26.0
	Total	319	100
Occupation category	Manufacture/Production	61	19.1
	Finance/Insurance	18	5.6
	Distribution	20	6.3
	Service	82	25.7
	R&D (research and development)	8	2.5
	IT/Information and Communications	17	5.3
	Others	113	35.4
	Total	319	100
Interest in auto maintenance and repair service	Very high	167	52.4
	Moderate	129	40.4
	Very low	20	6.3
	Not at all	3	0.9
	Total	319	100

**Table 3:** Analysis of trust and convergent validity of the measurement model.

Classification	Variable	Code	Standardized Factor Loading	Standard Error	T Value	CR	AVE	Cronbach α
Service relationship benefits	Confidence benefit (CB)	CB1	0.930	-	-	0.898	0.815	0.897
		CB2	0.874	0.043	23.121 ***			
	Social benefit (SB)	SB1	0.900	-	-	0.908	0.768	0.907
		SB2	0.842	0.049	20.634 ***			
		SB3	0.886	0.042	22.872 ***			
	Economic benefit (EB)	EB1	0.843	-	-	0.817	0.691	0.817
		EB2	0.819	0.060	15.770 ***			
Service trust (ST)		ST1	0.933	-	-	0.948	0.858	0.948
		ST2	0.920	0.034	29.685 ***			
Service satisfaction (SS)		SS1	0.928	-	-	0.948	0.838	0.912
		SS2	0.903	0.037	26.910 ***			
Customer engagement (CI)		CI1	0.894	-	-	0.912	0.825	0.904
		CI2	0.922	0.040	24.326 ***			
Long-term relationship orientation (RI)		RI1	0.954	-	-	0.967	0.907	0.966
		RI2	0.966	0.024	41.200 ***			
		RI3	0.936	0.028	35.383 ***			

(1) Measurement model fit: χ^2^(df) 240.81, p 0.0 DF 98, χ^2^/degree of freedom 2.457, Root Mean Square Residual(RMR) 0.400, Goodness-of-Fit Index (GFI) 0.919, Adjusted Goodness-of-Fit Index (AGFI) 0.874, Normal Fit Index (NFI) 0.962, Tucker Lewis Index (TLI )0.968, Comparative Fit Index (CFI) 0.977, Root Mean Square Error of Approximation (RMSEA) 0.068. (2) * *p* < 0.05, ** *p* < 0.01, *** *p* < 0.001.

**Table 4:** Discriminant validity.

	CR	AVE	CB	SB	EB	ST	SS	CI	RI
Confidence benefit (CB)	0.898	0.815	0.903						
Social benefit (SB)	0.908	0.768	0.620	0.876					
Economic benefit (EB)	0.817	0.691	0.584	0.709	0.831				
Service trust (ST)	0.948	0.858	0.79 *	0.691	0.638	0.926			
Service satisfaction (SS)	0.912	0.838	0.811	0.673	0.660	0.844	0.916		
Customer engagement (CI)	0.904	0.825	0.659	0.718	0.618	0.734	0.728	0.908	
Long-term relationship orientation (RI)	0.967	0.907	0.681	0.660	0.538	0.755 **	0.771	0.799	0.952

* *p* < 0.05, ** *p* < 0.01, *** *p* < 0.001.

**Table 5:** Hypotheses verification.

	Hypothesis	Standardized Factor Loading	Standard Error	CR (*p*)	Status of Acceptance	R2
H1	Confidence benefit → Service trust	0.622	0.06	10.952 ***	Accepted	0.797
H2	Social benefit → Service trust	0.249	0.071	3.331 ***	Accepted	
H3	Economic benefit → Service trust	0.095	0.087	1.234	Rejected	
H4	Confidence benefit → Service satisfaction	0.36	0.07	5.104 ***	Accepted	0.888
H5	Social benefit → Service satisfaction	0.029	0.06	0.427	Rejected	
H6	Economic benefit → Service satisfaction	0.167	0.072	2.449 *	Accepted	
H7	Service trust → Service satisfaction	0.458	0.071	6.048 ***	Accepted	0.681
H8	Service trust → Customer engagement	0.335	0.137	2.694	Rejected	
H9	Service satisfaction → Customer engagement	0.509	0.148	4.041 ***	Accepted	
H10	Service trust → Long-term relationship orientation	0.069	0.099	0.679	Rejected	0.775
H11	Service satisfaction → Long-term relationship orientation	0.335	0.114	3.09 **	Accepted	
H12	Customer engagement → Long-term relationship orientation	0.523	0.06	7.764 ***	Accepted	

(1) Structural model fit: χ^2^(df) 291.571, p 0.0, DF 104, χ^2^/degree of freedom 2.804, RMR 0.061, GFI 0.900, AGFI 0.853, NFI 0.954, TLI 0.960, CFI 0.970, RMSEA 0.075. (2) * *p* < 0.05, ** *p* < 0.01, *** *p* < 0.001.

**Table 6:** Direct and indirect effects analysis result.

Dependent Variable	Explanatory Variable	Direct Effect	Indirect Effect	Total Effect
Service Satisfaction	Service benefit (ST)	0.458	-	0.458
	Confidence benefit (CB)	0.360	0.285 **	0.645
	Social benefit (SB)	0.029	0.114 **	0.143
	Economic benefit (EB)	0.167	0.044	0.211
Customer engagement	Service satisfaction (SS)	0.509	-	0.509
	Service benefit (ST)	0.335	0.233 *	0.568
	Confidence benefit (CB)	-	0.536 **	0.536
	Social benefit (SB)	-	0.156 **	0.156
	Economic benefit (EB)	-	0.139 *	0.139
Long-term relationship orientation	Customer engagement	0.523	-	0.523
	Service satisfaction (SS)	0.335	0.266 *	0.601
	Service benefit (ST)	0.069	0.450 **	0.519
	Confidence benefit (CB)	-	0.539 **	0.539
	Social benefit (SB)	-	0.147 *	0.147
	Economic benefit (EB)	-	0.150 *	0.150

Note: * *p* < 0.05, ** *p* < 0.01, *** *p* < 0.001.

## References

[1] Jain N., Singh A., Kaushik K. (2019). Evaluating service quality in automobile maintenance and repair industry. Asia Pac. J. Mark. Logist..

[2] Rosenberg L.J., Czepiel J.A. (1984). A marketing approach for customer retention. J. Consum. Mark..

[3] Kodama F. (2019). Incessant conceptual/industrial transformation of automobiles. J. Open Innov. Technol. Mark. Complex..

[4] Kim Y.H., Ha K.S. (2015). Service factor is effect on revisiting for old people: Centering around mediator effect for feeding satisfaction. Asia Pac. J. Bus. Ventur. Entrep..

[5] Parvin S., Wang P., Uddin J. (2017). Assessing two consumer behavioural intention models in a service environment. Asia Pac. J. Mark. Logist..

[6] Chen C., Chen F. (2010). Experience quality, perceived value, satisfaction and behavioral intentions for heritage tourists. Tour. Manag..

[7] Leri I., Theodoridis P. (2019). The effects of the winery visitor experience on emotions, satisfaction and on post-visit behaviour intentions. Tour. Rev..

[8] Reichheld F.F., Sasser W.E. (1990). Zero defections: Quality comes to services. Harv. Bus. Rev..

[9] Gwinner K.P., Gremler D.D., Bitner M.J. (1998). Relational benefits in services industries: The customer’s perspective. J. Acad. Market. Sci..

[10] Palmatier R.W., Jarvis C.B. (2009). Bechkoff, J.R.; Kardes, F.R. The role of customer gratitude in relationship marketing. J. Mark..

[11] Hawaldar I.T., Ullal M.S., Birau F.R., Spulbar C.M. (2019). Trapping fake discounts as drivers of real revenues and their impact on consumer’s behavior in India: A case study. Sustainability.

[12] Quach T., Jebarajakirthy C., Thaichon P. (2016). The effects of service quality on internet service provider customers’ behaviour: A mixed methods study. Asia Pac. J. Mark. Logist..

[13] Reynolds K.E., Beatty S.E. (1999). Customer benefits and company consequences of customer-salesperson relationships on retailing. J. Retail..

[14] Ulaga W. (2003). Capturing value creation in business relationships: A customer perspective. Ind. Mark. Manag..

[15] Conze O., Bieger T., Laesser C., Riklin T. (2010). Relationship intention as a mediator between relational benefits and customer loyalty in the tour operator industry. J. Travel Tour. Mark..

[16] Reimer A., Kuehn R. (2005). The impact of service-scape on quality perception. Eur. J. Mark..

[17] Shankar A., Jebarajakirthy C. (2019). The influence of e-banking service quality on customer loyalty: A moderated mediation approach. Int. J. Bank Mark..

[18] Lie D., Sudirman A., Butarbutar M. (2019). Analysis of mediation effect of consumer satisfaction on the effect of service quality, price and consumer trust on consumer loyalty. Int. J. Sci. Technol. Res..

[19] Koritos C., Koronios K., Stathakopoulos V. (2014). Functional vs relational benefits: What matters most in affinity marketing?. J. Serv. Mark..

[20] Unidha M. (2017). The effect of service quality on trust and loyalty for giant customers in Malang city. Arab. J. Bus. Manag. Rev..

[21] Darkhantuya S., Park Y.S., Kim Y.S. (2017). The effect of relationship benefits on customer citizenship behavior mediated by gratitude and customer satisfaction. J. Korea Serv. Manag. Soc..

[22] Kang S.M., Kim H.J. (2018). Effects of relationship benefits on customer satisfaction and long-term relationship orientation: Focused on credit unions. Asia-Pac. J. Bus. Ventur. Entrep..

[23] Izogo E.E. (2015). Customers’ service quality perception in automotive repair. Afr. J. Econ. Manag. Stud..

[24] Pantouvakis A., Patsiouras C. (2016). Exploring the role of leadership style on the service quality customer satisfaction link. Int. J. Qual. Serv. Sci..

[25] Hennig-Thurau T., Gwinner K.P., Gremler D.D. (2002). Understanding relationship marketing outcomes: An integration of relational benefits and relationship quality. J. Serv. Res..

[26] Crosby L.A., Evans K.A., Cowles D. (1990). Relationship quality in services selling: An interpersonal influence perspective. J. Mark..

[27] Mohr J., Spekman R. (1994). Characteristics of partnership success: Partnership attributes, communication behavior, and conflict resolution techniques. Strateg. Manag. J..

[28] Storbacka K., Standvik T., Gronroos C. (1994). Managing customer relationships for profit: The dynamics of relationship quality. Int. J. Serv. Ind. Manag..

[29] Harris L.C., Ezeh C. (2008). Servicescape and loyalty intentions: An empirical investigation. Eur. J. Mark..

[30] Van Doorn J., Lemon K.N., Mittal V., Nass S., Pick D., Pirner P., Verhoef P.C. (2010). Customer engagement behavior, Theoretical foundations & research directions. J. Serv. Res..

[31] Calvert G.A., Pathak A., Ching L.E.A., Trufil G., Fulcher E.P. (2019). Providing excellent customer service is therapeutic: Insights from an implicit association neuromarketing study. Behav. Sci..

[32] Brodie R.J., Hollebeek L.D., Juric B., Ilic A. (2011). Customer engagement, conceptual domain, fundamental propositions, and implications for research. J. Serv. Res..

[33] Verhoef P.C., Reinartz W.J., Krafft M. (2010). Customer engagement as a new perspective in customer management. J. Serv. Res..

[34] Izogo E.E., Ogba I.E. (2015). Service quality, customer satisfaction and loyalty in automobile repair services sector. Int. J. Qual. Reliab. Manag..

[35] Yuen K.F., Thai V.V. (2015). Service quality and customer satisfaction in liner shipping. Int. J. Qual. Serv. Sci..

[36] Shin B.S. (2014). The influence of consumers perception toward relationship benefit and fairness on long-term relationship orientation with franchise store: Focus on hair service. Korea Entrep. Soc..

[37] Jeon S.B., Jeong B.G., Lee J.S. (2013). The effect of relationship benefits on customer satisfaction and loyalty in the beauty salons. J. Korean Soc. Cosmetol..

[38] Kelley H.H., Thibaut J.W. (1978). Interpersonal Relationship: A Theory of Interdependence.

[39] Johnson M.D., Herrmann A., Huber F. (2006). The Evolution of Loyalty Intentions. J. Mark..

[40] Meadows M., Dibb S. (2012). Progress in customer relationship management adoption: A cross-sector thesis. J. Strateg. Mark..

[41] Itani O.S., Kassar A.N., Loureiro S.M.C. (2019). Value get, value give: The relationships among perceived value, relationship quality, customer engagement, and value consciousness. Int. J. Hosp. Manag..

[42] Fischmann G., De Witte H., Sulea C., Iliescu D. (2018). Qualitative job insecurity and in-role performance: A bidirectional longitudinal relationship?. Eur. J. Work Organ. Psychol..

[43] Nora L. (2019). Trust, commitment, and customer knowledge: Clarifying relational commitments and linking them to repurchasing intentions. Manag. Decis..

[44] La S., Choi B. (2012). The role of customer affection and trust in loyalty rebuilding after service failure and recovery. Serv. Ind. J..

[45] Flavián C., Guinalíu M., Gurrea R. (2006). The role played by perceived usability, satisfaction and consumer trust on website loyalty. Inf. Manag..

[46] Lai T.L. (2004). Service quality and perceived value’s impact on satisfaction, intention and usage of short message service (SMS). Inf. Syst. Front..

[47] Huang Y., Moon T. (2018). Influence of car sharing service on consumers’ continuous usage intention in China. J. Internet Electron. Commer. Res..

[48] Chahal H., Kumari N. (2012). Consumer perceived value: The development of a multiple item scale in hospitals in the Indian context. Int. J. Pharm. Healthc. Mark..

[49] Wang X.W., Cao Y.M., Park C. (2019). The relationships among community experience, community commitment, brand attitude, and purchase intention in social media. Int. J. Inf. Manag..

[50] Ruiz-Molina M.E., Gil-Saura I., Berenguer-Contrí G. (2009). Relational benefits and loyalty in retailing: An inter-sector comparison. Int. J. Retail Distrib. Manag..

[51] Song K.S., Lee N.Y. (2017). Study on social enterprise customer’s relational benefits impact on repurchase intention and intention to recommend: Focusing on the mediating effect of customer-company identification. J. Hum. Resour. Manag. Res..

[52] Sirdeshmukh D., Singh J., Sabol B. (2002). Consumer trust, value, & loyalty in relational exchanges. J. Mark..

[53] De Wulf K., Odekerken-Schröder G., Iacobucci D. (2001). Investment in consumer relationships: A cross- country and cross-industry exploration. J. Market..

[54] So K.K.F., King C., Sparks B. (2014). Customer engagement with tourism brands scale development & validation. J. Hosp. Tour. Res..

[55] Ganesan S. (1994). Determinants of long-term orientation in buyer-seller relationship. J. Mark..

[56] Jahanshahi A.A., Gashti M.A.H., Mirdamadi S.A. (2011). Study the effects of customer service and product quality on customer satisfaction and loyalty. Int. J. Hum. Soc. Sci..

[57] Yoon K.H., Kim B.Y., Eom J.G. (2019). The effects of job crafting on career success of multinational corporations’ employee. J. Asian Financ. Econ. Bus..

[58] Yoon D.Y., Kim B.Y. (2019). The effects of core self-evaluation factors of female salesperson on sales performance. J. Distrib. Sci..

[59] Gallagher D., Ting L., Palmer A. (2008). A journey into the unknown; taking the fear out of structural equation modeling with AMOS for the first-time user. Mark. Rev..

[60] Wu S.H., Huang S.C.T., Tsai C.Y.D., Lin P.Y. (2017). Customer citizenship behavior in social networking sites: The role of relationship quality, identification, and service attributes. Internet Res..

[61] Bei L.T., Chiao Y.C. (2001). An integrated model for the effects of perceived product, perceived service quality, and perceived price fairness on consumer satisfaction and loyalty. J. Consum. Satisf. Dissatisf. Complain. Behav..

[62] Famiyeh S., Asante-Darko D., Kwarteng A. (2018). Service quality, customer satisfaction, and loyalty in the banking sector: The moderating role of organizational culture. Int. J. Qual. Reliab. Manag..

[63] Andaleeb S.S., Basu A.K. (1998). Do warranties influence perceptions of service quality: A study of the automobile repair and service industry. J. Retail. Consum. Serv..

[64] Park J.O., Park I.S., Yang C.S. (2010). The effects of service quality of auto maintenance and repair services on customer satisfaction and customer loyalty. J. Korea Ser. Manag. Soc..

[65] Oláh J., Yusmar A.H., Máté D., Novotny Á., Popp J., Lakner Z., Kovács S. (2019). A trust approach to the financial performance of information and communications technology enterprises. Pol. J. Manag. Stud..

[66] Pakurár M., Benedek S.A., Popp J., Magda R., Oláh J. (2019). Trust or doubt: Accuracy of determining factors for supply chain performance. Pol. J. Manag. Stud..

